# Using telepresence robots as a tool to engage patient and family partners in dementia research during COVID-19 pandemic: a qualitative participatory study

**DOI:** 10.1186/s40900-023-00421-w

**Published:** 2023-03-23

**Authors:** Lillian Hung, Charlie Lake, Ali Hussein, Joey Wong, Jim Mann

**Affiliations:** School of Nursing, University of British Columbia, T201-2211 Wesbrook Mall, Vancouver, BC V6T 2B5 Canada

**Keywords:** Telepresence robots, Patient and public involvement, COVID-19, Virtual research, Long-term care

## Abstract

**Background:**

Long-term care (LTC) settings have been disproportionately affected by the COVID-19 pandemic; it is important to address unmet needs and explore practical strategies for supporting LTC residents and staff. The involvement of patient partners and family community members in research planning, implementation and evaluation is the basis of Patient and Public Involvement approach and has been challenging during the COVID-19 pandemic, as visitation restrictions have created barriers to conducting research in healthcare settings. Innovative methods and tools are needed for conducting participatory research. This study investigated the use of telepresence as innovative tools for participatory research based on three projects conducted with patient and family partners during the COVID-19 pandemic.

**Methods:**

The data source includes (a) team reflective discussions, (b) weekly meeting notes, (c) field notes, and (d) interviews with ten researchers. We applied purposive sampling to select ten researchers who used a telepresence robot to conduct research in British Columbia, Canada. Semi-structured one-to-one interviews were conducted via Zoom virtual meetings. Patient and family partners contributed to team analysis to identify themes.

**Results:**

Analysis of the data produced five themes: (1) Research Enabler, (2) User-Friendly Technology, (3) Increased Engagement, (4) Lack of Infrastructure and Resources, and (5) Training and Technical Obstacles. Based on the results, we propose “ROBOT”—an acronym for five actionable recommendations to support the use of telepresence robots for research. The ROBOT recommendations represent: Realign to adapt, Organize with champions, Blend strategies, Offer timely technical assistance, and Tailor training to individual needs.

**Conclusions:**

This study offers practical insights into the use of telepresence robots as a safe and innovative tool for conducting remote research with people with dementia, even in times of restricted access, as with COVID-19. Future research should apply more creativity and flexibility in adopting technology to expand possibilities for involving people with dementia in research.

## Abstract (summary)

It is important to address unmet needs and explore practical strategies for supporting long-term care (LTC) residents and staff who have been disproportionately affected by the COVID-19 pandemic. Involving patient partners and family community members in research planning, implementation, and evaluation is crucial for understanding the challenges faced by LTC residents. However, the restrictions on visitation and staff shortages during the pandemic have made this difficult. This study investigated the use of telepresence robots as innovative tools for participatory research. We interviewed ten researchers who used telepresence robots to conduct remote research in British Columbia, Canada. Semi-structured one-to-one interviews were conducted via Zoom meetings and analyzed to find common themes. Based on the analysis of the interviews and our reflection, five themes were found: (1) Research Enabler, (2) User-Friendly Technology, (3) Increased Engagement, (4) Lack of Infrastructure and Resources, and (5) Training and Technical Obstacles. Based on these findings, we propose “ROBOT”—an acronym for actionable recommendations to support the use of telepresence robots for research. These recommendations include: Realign to adapt, Organize with champions, Blend strategies, Offer timely technical assistance, and Tailor training to individual needs. This study provides insights into the use of telepresence robots as safe and innovative tools for conducting research with patient and family partners remotely, even in times of restricted access, such as during the COVID-19 pandemic.

## Introduction

Long-term care (LTC) settings around the globe have been disproportionately affected by the COVID-19 pandemic, with LTC homes in Canada being first locked down in March 2020. In light of new public health measures and visitation restrictions, staff shortages and burnout [1], limited social connection, and increased isolation [2], there is a great demand to explore the needs of older adults and staff in LTC and strategies to support them. “Nothing for us, without us,” having residents’ and staff voices in research for long-term care is crucial to understand their experiences and complex conditions fully.

Meanwhile, the unprecedentedly high number of public health orders, uncertainty, and intense staff shortages have created physical and psychological challenges and barriers to conducting research in healthcare settings [3]. Safety concerns and public health orders have restricted researchers from entering the LTC sites for data collection, the collaboration of implementation with the site, and the engagement of staff and residents. Many researchers are forced to change from face-to-face data collection to other virtual alternatives [4]. The transition from in-person to virtual settings delayed project timelines from what was initially planned [5]. Furthermore, observing participants’ non-verbal cues, e.g., facial expressions, minor gestures, and actions, is challenging without in-person research [6, 7]. Poor internet connection in some regions led to pauses, interview disruptions, and poor audio and visual delivery [6]. Even for situations where in-person data collection was allowed, masks impacted building a rapport and trusting relationship between researchers and participants during an interview. Personal Protective Equipment (e.g., facial shield and mask) contributed to a substantial loss of visual and auditory cues for participants with hearing impairment [8]. In addition, researchers may face organizational barriers as some LTC homes have staff shortages that impede technological implementation [9].

Patient and Public Involvement (PPI) in research, which is widely promoted internationally in countries such as Canada [10], the UK [11] and the US [12], has faced challenges during the COVID-19 pandemic. PPI refers to research that is conducted ‘with’ patient partners and the public, rather than solely being done 'to', 'about' or 'for' them. This approach means that persons with relevant lived experience (patient and family partners) have a significant role in shaping how research is designed, conducted, and disseminated [13]. The four best practice principles for PPI are: “(1) involve the right people, (2) involve enough people; (3) involve those people enough; (4) describe how it helps” [14]. It is important to integrate the perspectives of individuals with lived experiences into every stage of the research project, from research planning to data collection and evaluation. However, under the socially distanced pandemic environment, patient partners from marginalized and vulnerable groups experience further exclusion, disadvantages, and struggles in health, economic and social conditions—for example, limited travel and challenging access to healthcare and technology [15]. There is a strong need to use technology to support connection and collaboration with patient partners in research [15].

Despite numerous safety concerns and barriers, challenges bring opportunities. Alternatives and recommendations for research design, tools, and technology for conducting research emerged to support the research process [6]. Phone calls, photovoice, video teleconferencing, text-based mediums, digital diaries, and Zoom calls have been used in interviews and focus groups [4, 6, 15]. These tools have their strengths and drawbacks. For example, using phones does not require learning new technologies or using the Internet. However, interviewers cannot observe participants’ non-verbal cues through phone calls.

Moreover, research showed that researchers take more time to build trust and rapport with interviewees via phone than face-to-face [15]. Teleconferencing and Zoom calls allow video conversations and observations. Still, there will be a need for Internet support and staff on-site to set up calls and assist with technical issues encountered [15].

**Fig. 1:**
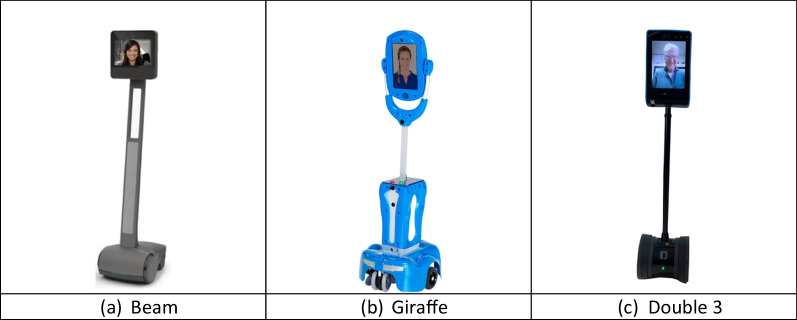
**a** Beam [27]; **b** Giraffe [28]; **c** Double 3

Besides facilitating research processes, technologies emerged to support social connections for residents in LTC. Telepresence robots are one of them. These robots are mobile communication devices that can enable virtual video and audio communication. They have various functions with their cameras, microphones, and loudspeakers, e.g., mobility of 360°, height alterations, recording, and video and audio functions [16]. Examples of telepresence robots are Beam, Giraff, and Double 3 (Fig. 1). People use telepresence robots in the business and educational fields [17]. Recently, research has been conducted using telepresence robots in geriatric care settings for social connections [16, 17]. Researchers have found that telepresence robots enable the feeling of the person's presence remotely via the robot [18–21]. Moreover, telepresence robots are easy for people living in LTC with physical frailty and cognitive impairment [18, 22–25]. Residents do not need to learn how to control the robots as they are remotely controlled by the person calling [19, 26].

### The research team

Our research team consists of an academic researcher, 20 student trainees from both undergraduate and graduate levels, and people with lived experience (three people living with dementia and three family caregivers). By involving the right people, specifically patient and family partners (who are living well in the community), we ensure that our research is designed in a manner that is acceptable and beneficial for participants, such as long-term care residents. Also, our patient and family partners contribute to better communication of the research to the participants and public by serving as ambassadors and spokesperson for our studies.

Our research team has been studying the use of telepresence robots (Double 3) in LTC to facilitate virtual connections between residents and their families. We involve patient and family partners as co-leads and co-researchers in all our projects, but during the pandemic, we faced challenges in engaging them actively. We have used telepresence robots as an innovative tool to overcome these challenges and enable patient and family partners to continue to contribute to research work. For example, JM (patient partner), a project co-lead has used the robot to conduct conversational interviews, observation, and focus groups in LTC. Other patient and family partners have applied the telepresence robot to conduct workshops and interviews in LTC, attend remote research meetings, and provide knowledge dissemination. Yet, telepresence robots have not been documented as a tool for research from a recent literature review on participatory research during the COVID-19 pandemic [15].

The purpose of this paper is to report the challenges, benefits, and implications of using telepresence robots to support patient and family partners to contribute to research. Based on our lessons learned, we hope to spark further conversation on this innovative approach and encourage more active engagement of patient and family partners in research.

## Methods

### Study design and location

A qualitative descriptive design was selected to capture rich details of researchers’ experiences using telepresence robots to conduct interviews and workshops in three research projects. Table 1 describes the details of each research project and how telepresence robots were used in them.

**Table 1:** Descriptions of the three research projects that used telepresence robots as a tool

Happy2Eat	This Participatory Action Research (PAR) project explores practical strategies to improve the dining experience of LTC residents with dementia. As data was generated through conversations with residents, families, staff, and leaders, the telepresence robots were used to visit the site and have in situ discussions with LTC residents
Overcoming Loneliness	This project focused on learning more about residents’ experiences of loneliness in LTC homes as impacted by the COVID-19 pandemic and developing strategies to overcome challenges. Data was collected through one-on-one interviews with residents using telepresence robots for private conversations in their rooms
WhatMatters	WhatMatters is a digital care plan mobile app developed in collaboration with the stakeholders in co-design workshops. The telepresence robots were used in the workshops virtually as part of the design process

### General procedures of the above research conducted with a telepresence robot

**Fig. 2:**
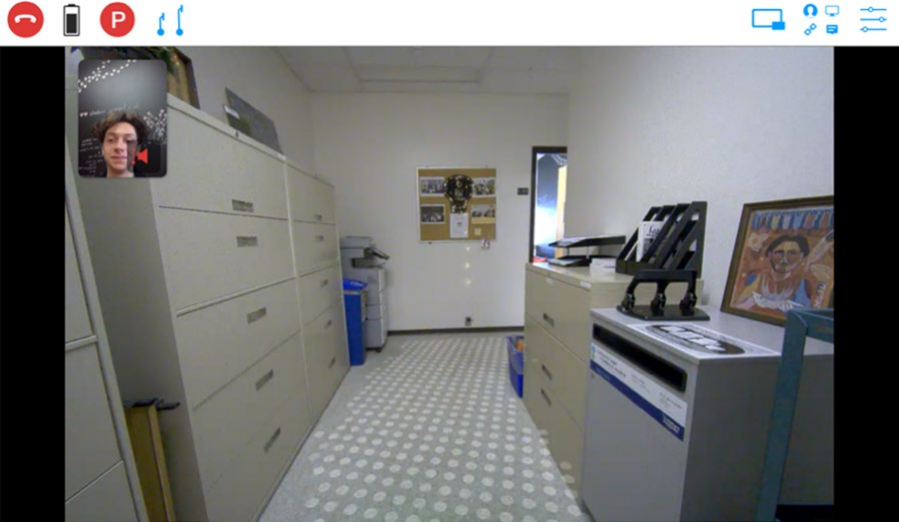
Sample screen view for interviewers

The three projects were all conducted in five LTC homes in British Columbia, Canada. All LTC homes were non-profit and in large size. In general, the researchers asked the LTC residents open-ended questions (e.g., what is your experience of…? What do you wish to have to support you?) The researchers also followed the lead of residents and encouraged them to tell stories about what mattered to them. The interviews were conversational. The resident stories generated rich data. Before each session, the researchers asked the staff to place the robot in the resident's room. After that, the staff left the room to allow privacy for the interview. With the specific link for the scheduled interview sent by our technical team member, the interviewer could “call in” the telepresence robot using laptops, smartphones, or tablets. The interviewers’ faces would appear on the robot’s screen once they called in virtually. The interviewers could see the resident in the room (Fig. 2). As telepresence robots give a broader view (see Fig. 2) compared to other video teleconferencing interfaces such as Skype and Zoom, the visual access allows observation of the resident interacting with the environment in real-time. With residents' consent, the interviews were recorded via the system of the robots.

### Data collection

The data source for this paper was generated from (a) team reflective discussions, (b) weekly meeting notes, (c) field notes, and (d) interviews with ten researchers from a large team. In our weekly research meetings, patient partners were able to actively contribute to the planning and problem-solving of any issues that emerged during the research. This was particularly valuable for our project co-lead, JM, as he provided crucial guidance, direction, and decision-making for the research team. We also made sure to incorporate team reflection into these meetings, as shown in Table 2, which includes reflexive questions and the actions we took to ensure reflexivity in the three research projects mentioned above.

**Table 2:** Reflection and action

Reflexive questions	PPI principles	Action examples
1. How did the research team ensure involvement of the right people in the three projects?	Principle 1: Involve the right people	Involved patient and family partners with relevant and direct experience of dementia and LTC
2. Did the team involve enough patient and family partners in the three projects?	Principle 2: Involve enough people	Involved people with diverse backgrounds and experiences (3 patient partners, 3 family partners) to provide a reasonable breadth and depth of views on the LTC issues
3. Did the team involve patient and family partners enough in all three projects?	Principle 3: Involve those people enough	Involved early, e.g., JM served as the co-lead, MG, LJ, LW, AB, SD were involved in research grant preparation, study planning, shared decision-making on roles and responsibilities, data collection and analysis, co-authorship in reporting research in conferences, publication, as well as served as spokespersons in media communications
4. How did patient and family partners help/contribute to all three research projects?	Principle 4: Describe how it helps	All patient and family partners helped identify research questions and outcome priorities in preparing the proposal to ensure relevance and acceptance Contributed to problem solving in weekly research meetings Contributed to data collection by conducting interviews and team analysis Contributed to a rewarding intergenerational learning experience for trainees (university students) Contributed to knowledge translation activities, e.g., speaking to the TV news, public videos

We conducted a self-study project in the IDEA (Innovation in Dementia and Aging) Lab in British Columbia, Canada. We used a purposive sampling method to select ten researchers with various backgrounds in race, age, sex and gender, as well as disciplines. We included researchers who used the robot as a tool for the three research studies described above during the COVID-19 pandemic. There were no specific exclusion criteria. We conducted semi-structured individual interviews with the ten researchers between September and December 2021. See Table 3 for the demographic information of the researchers. The interviewees belong to the same research lab. One interviewee (JM) is an experienced researcher with lived experience of dementia and co-author of this paper. JM is listed as an author as he is the co-lead of this Telepresence Robot project and has contributed substantially towards this paper (i.e., design, planning, data collection, analysis, and dissemination).

**Table 3:** Demographic information of the researchers interviewed (N = 10)

*Gender:*	
Male	1 (10%)
Female	9 (90%)
Age range	22–81
*Racial background*	
Caucasian	3 (30%)
Asian	7 (70%)
*Discipline:*	
Nursing	3 (30%)
Science	2 (20%)
Design	2 (20%)
Patient/family partner	3 (30%)

In the interviews we asked two questions: What was the researcher’s experience in using the telepresence robot in conducting research? What were the lessons learned—what worked well and what kind of practical lessons were learned to overcome challenges? Two authors (CL and AH) conducted individual interviews. The interviews were conducted virtually via Zoom meetings, digitally recorded and transcribed verbatim. Each interview lasted about 30 min.

### Data analysis

Data analysis involved inductive methods: thematic analysis [29]. The first author is an academic professor who guided data collection and analysis procedures. Authors CL and AH (trainees) performed the analysis in three steps. First, CL and AH read the data in the interview transcriptions, team weekly meeting notes, field notes, and reflection notes to gain familiarity with the content. Second, codes and patterns were searched across the data and identified initial themes. Then patient partner co-author, JM, discussed the results with the whole team. He contributed to team analysis by highlighting the key results and their relevance to meaningful involvement in PPI. For example, he challenged the team about the assumptions of involving people with dementia in research, which led to a productive discussion about how persons with dementia can play an active role in all aspects of research. He also inquired about how the robot enabled him to be a research co-lead during the pandemic. The team discussion brought further insights into the meanings of his contribution in using the robot as a tool to conduct data collection—how it challenged the stigmatization associated with dementia and demonstrated the valuable contributions patient partners can make to improve research. The team analysis process involved going back and forth between the data and team discussion. Finally, all authors worked together in the final step of the analysis to gain analytic consensus and complete the last write-up. This step focused on reviewing and refining the final themes. Here we present our empirical insight about using a telepresence robot, Double 3, as a research tool to support participatory research during the public health crisis of COVID-19.

### Rigour

We used a reflexive approach to reflect on our role as individual researchers and critically examined how we made sense of the data individually and collectively as a team. To ensure credibility, initial themes were discussed by all team members, a process that generated iterative cycles of refinements. Team reflexivity was embedded in all our meetings throughout all research stages. For dependability, we asked critical questions about our intentions and compared assumptions. For example, our patient partner (JM) often reminded us how our values and beliefs might inform and shape our decision-making and approaches. In the concurrent data collection and analysis process, we asked what worked well and what did not, why, and how. For confirmability, we wrote research notes and kept an audit trail. The team discussion informed subsequent data collection. Including a patient partner in the research team helped provide a lived experience perspective and kept every step of our work transparent. As our experience can be helpful to other researchers, we documented detailed descriptions of the research procedures to support transferability. The research team also ensured that the processes of the study were informed logically by conducting a comprehensive review of the existing literature to address gaps in the literature.

## Results

**Fig. 3:**
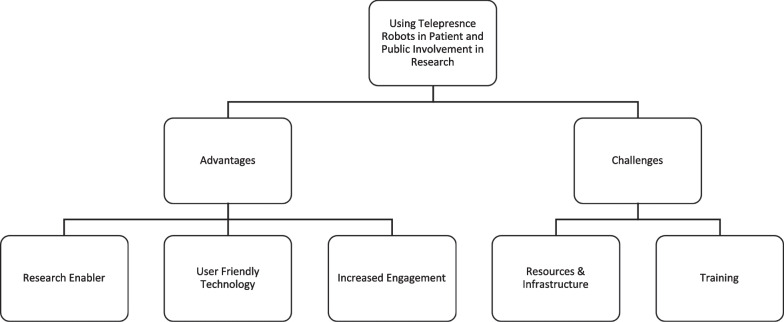
Summary of Themes and Subthemes

Our reflection and analysis indicated advantages and challenges with the use of telepresence robots as a tool to support patient involvement in research. Based on the results, we identified five themes: (1) Research Enabler, (2) User-Friendly Technology, (3) Increased Engagement, (4) Lack of Infrastructure and Resources, and (5) Training and Technical Obstacles (Fig. 3).

### Advantages

#### Use as a research enabler

The first and main benefit of introducing this novel technology into the research field was that it allowed PPI to take place that would otherwise be impossible during a pandemic that restricted in-person visits in LTC. The researcher interviewees (a family partner) reported that they successfully used the telepresence robot in LTC homes to conduct interviews with residents, which served as a viable alternative to in-person interviews. One concern when using tablets and smart phones to perform research remotely was that staff assistance was required to support the LTC residents with physical and cognitive disabilities during the interview, impacting the resident’s privacy due to the staff's presence. The telepresence robots enabled the researchers to hear the direct voices of residents living in LTC without staff’s presence. This was especially important for high-risk communities such as LTC homes.

Telepresence robots facilitated safe and active involvement of patient partners, particularly older individuals, in the research process. They enabled patient and family partners to remotely visit study sites, participate in research meetings, and interact with resident participants in conversations. Despite pandemic restrictions, telepresence robots allowed patient and family partners to virtually attend “in-person” meetings. They also provided more freedom compared to video conferencing platforms like Zoom, as the patient partners could move around the room and join the conversations in a more natural way.

During infection outbreaks and crisis times in the pandemic, telepresence robots play a significant role in data collection at the sites by patient and family partners. They continued to conduct interviews and observations via the robot to understand the experience of LTC residents. This allowed our other patient partners to actively drive our research instead of being limited by visitation restrictions. However, other alternatives exist that enable virtual research instead of in-person interviews; the researcher interviewees (R2 and R4) explained other virtual communication interfaces, such as Zoom or Skype, would require staff to set up a device and assist the resident in operating a computer or a tablet during the interview. Staff assistance for research was very limited or inaccessible during the pandemic in LTC due to staffing shortages. Also, the staff’s presence could take away the privacy of the interview conversation. Therefore, the telepresence robot has the relative advantage of saving staff assistance time and enabling residents to be interviewed. All the researcher interviewees repeatedly underscored the convenience of having the robot as a tool for research. As R6 remarked, “I can easily and safely conduct the research through the robot without taking the risk of entering the care home. I can talk to the residents; I can see and move around in the dining room. I can ask staff questions while they are interacting with the residents. The conversations felt normal, as if I was there. It was handy”. The mobile robot allowed the research activities to take place in the dynamic environment of the LTC dining room, providing authentic experiences like in-person interviews. Patient and family partners shared their perspectives and experiences in team meetings and contributed to the team's analysis.

Telepresence robots also enabled patient partners to get involved in knowledge dissemination. Patient partner JM even used the robot to remotely attend an interview with the TV media, where he was able to walk with the journalist and share his insights on how telepresence robots helped him visit long-term care sites remotely. This advanced knowledge translation to a “different form of presence” through the patient partners’ involvement.

#### User-friendly technology for patient partner researchers

At the beginning, patient and family partners expressed feeling intimidated by the idea of having to operate a robot and felt unsure of its usefulness. However, after receiving training and practicing, they quickly became comfortable with its use. The training provided by students also helped to foster intergenerational relationships within the team. The interaction between young students and older adult patient and family partners related to the use of the robot challenged the stereotypes and biases around dementia and ageism in technology use. The patient and family partners demonstrated that they were able to learn and operate the robot in research, which brought them a sense of confidence, accomplishment, and pride.

Furthermore, it was easier to reach the resident participants through a direct link with the robot, compared to scheduling a Zoom call through a staff (R4, R5). Many reported that they were also able to collect and store more information than if the interviews were in-person through recording the interviews (R1–3). They explained that the robot provided an all-in-one device to conduct the research, record the interviews, and store the data. They did not need to gather speakers, recorders, and computers. They also did not need to worry about putting on Personal Protective Equipment (PPE) or infection policy and procedures in remote research. The robot also allowed real-time adjustment for sound quality. According to researchers 6 and 7 (R6–7), the full-face expression and good audio volume led to a more natural flow of conversation and improved data collection. Often, the robot was cited as “the closest alternative to an in-person meeting that is currently available”; for research that is ideally performed in person, the robot was often mentioned by interviewers (R1–3, R6–8) as the preferred alternative when in-person interviews were unavailable: “It was amazing how they looked at me as if I was really there. They have no problems with sharing their stories with me” (R7). Notably, patient and family partners (interviewers) reported that the resident participants were comfortable speaking with them through the robot. The robot created an informal and relaxed atmosphere for conversation. This could be attributed to several factors. Firstly, patient and family partners (with lived experience with dementia) employed an appropriate approach to interact with residents during the interviews. Secondly, conducting the interviews in the residents' living environment likely made them more at ease. Lastly, compared to being interviewed in front of a computer screen, the rolling robot may have felt less intrusive or formal to the resident participants, making for more comfortable conversational interviews.

Many researchers also discussed the benefits of the mobility of the robot for research, having an additional option to follow residents around and observe their interactions with people and the physical environment. For example, R6 described how the robot In the Happy2Eat project allowed him to conduct focus group conversations with a few staff and observe how residents were being assisted to eat in the dining room. In the WhatMatters project, resident participants showed off the materials in their surroundings and took the researchers to see knickknacks in their own room. The robot expanded opportunities to interact with residents and allowed engaged observation with staff and residents in the field that would otherwise be impossible with a stationary/handheld device (R7, R9).

#### Increased engagement with residents

The interviews held through the telepresence robots were found to be more engaging compared to alternative virtual interview methods such as Skype and phone calls (R1–7, R9). Despite the telepresence robots being a non-traditional and unfamiliar interview method to residents, the researcher (R4) said none of the residents she interviewed seemed uncomfortable with the robots. “In fact, all residents responded positively to the robot, often seeming excited about the interviews. One resident even told me the robot was the next best thing to a real person” (R4). These positive experiences were crucial for patient and family partners to feel a sense of purpose and meaningful contributions to the research and community. In our field note and team reflection, patient and family partners often said, “I am glad that I was able to help. I want to feel useful for the research.”

In the WhatMatters project, two researchers (R5, R7) said “many residents took advantage of the wide view of the robot to show off objects in their room and told us stories about them. That was very helpful in our co-design workshop to gain an understanding of what matters to them and their lives”. In this way, seeing more of the environment contributed positively to the engagement of residents in the interviews. Residents often enjoyed the interviews so much that “they lost track of time, being so engaged in the conversations” (R5).

Researcher (R5) further discussed how the body language in their calls enhanced residents’ responses, as the robots enabled a wide view of the environment. A researcher (R7) suggested that the robot allowed the resident to take an active role in deciding what should be included in the research as the resident could take the lead to move through the space and show more to researchers, compared to an interview through a platform such as Zoom. Another researcher (R8) added that the controlled view of the researcher contributed positively to the interview. “As the researcher could see what their own camera captured and displayed to residents, they had more control over the body language they presented. This allowed this researcher to ensure that all body language seen by residents, such as facial expressions, was positive and non-judgemental” (R8).

Telepresence robots also provided opportunities for patient partners to be creative in designing engagement plans. During a discussion on how the team could improve engagement with residents and staff in long-term care facilities using telepresence robots, a patient partner suggested dressing up the robots for special occasions such as Halloween, Christmas, and Father's Day at the LTC sites. The patient and family partners were actively involved in team discussions on planning and implementing the idea.

### Challenges

#### Lack of infrastructure and resources

Many of the interviews suffered from a poor connection, leading to interruptions in the call between the resident and the interviewer. This was not surprising, as Wi-Fi standards and availability are inconsistent between and within LTC sites [30]. As Wi-Fi was not always adequate in resident rooms, “interviews a few times had to be held and scheduled in other rooms, which placed residents in unfamiliar environments” (R6). “Scheduling challenge sometimes arose. Planning interviews can be difficult because I need a staff to bring the robot to the resident. Finding a staff member to help is not always easy” (R4).

#### Training and technical obstacles

Another challenge in using telepresence robots for interviews was their novelty and the lack of familiarity of interviewers, residents, and LTC staff with this technology; because this technology was so new, some researchers were initially uncertain about using it. The robot first had to be introduced to a site before interviews could be held, with training provided to staff at LTC homes to ensure the robot would be properly handled and taken care of. Furthermore, interviewers had to be trained briefly on how to connect to the robot and use many of its features. Feedback in our interviews showed that researchers who were provided more training with the robot felt more comfortable and viewed their interviews as being more successful (R1–3, R5, R6, R9).

Training of both these groups required time before the interviews could be held and often had scheduling conflicts that slowed down the period required to hold interviews at a specific site. Additionally, other interview methods, such as phone interviews or virtual video interviews through platforms such as Zoom, may have been preferred by interviewers, residents, or staff due to their familiarity with the methods (R4–6). In this case, the novelty and lack of familiarity with telepresence robots was also an obstacle to performing research.

It was important for training to be given to alleviate staff and interviewer concerns. For example, LTC staff often expressed concerns about the privacy of residents being interviewed, often worrying about the telepresence robot recording even in cases where the robot was off or not in use. Training provided to the sites helped alleviate concerns such as these, allowing our research to gain more support from staff. It was important for accommodations to be made to ensure support from staff; to help with privacy concerns, we provided covers for the robots to cover any cameras when the robots were not in use. This allowed staff to feel confident that their privacy was protected with the technology. This training and support were, therefore, necessary and could be perceived by staff as extra burden adding to their already exhausting heavy workload in LTC. Privacy risks and concerns such as these were present when implementing the telepresence robots for use in research. Our recently published paper [31] reported the ethical concerns in detail and provided suggestions for mitigating these issues.

## Discussion

The COVID-19 pandemic has negatively impacted healthcare research due to in-person gathering restrictions [32–34]. Remote research has become increasingly ubiquitous. Yet, there has not been any investigation into using telepresence robots for participatory research. In this article, we reflected on the experience of using telepresence robot to involve patient and family partners in dementia research. Other scholars have shown benefits to using telepresence robots in LTC homes to help facilitate care for patients with dementia, finding that the telepresence robots may have increased the presence and engagement of family members in LTC [22, 25, 26, 37]. However, these studies have primarily focused on using telepresence robots for family calls rather than investigating telepresence robots as a research tool to enable PPI in research. Many other researchers have also had to convert research to virtual platforms due to COVID restrictions [35, 36]. From their experiences, they reported having to modify their research design slightly to fit the new circumstances brought forth by the pandemic restrictions [35, 36]. These studies did not use telepresence robots but still, show that virtual research may improve research access if appropriately implemented. It is clear from these studies that there is a strong need for further exploration of helpful virtual research tools to enable PPI. Future research should provide more innovative strategies to support meaningful involvement of patient partners in remote research.

### Effectively performing participatory research using telepresence robots

To help summarize how the telepresence robot was used in research and how it can be used in the future, we developed an acronym to offer actionable insights on how best to utilize the robot for research. The acronym ROBOT suggests the following: Realign to adapt, Organize with champions, Blend strategies, Offer timely technical assistance, and Tailor training to meet individual needs.

#### Realign to adapt

The research team must be flexible in their approach and adapt their methods as necessary for the specific location. Input from academic researchers, patients, families, long-term care residents, and staff should be considered and used to realign with research goals. While the telepresence robot is innovative, more needs to be understood about how to overcome technical issues and logistical challenges in its use for research. Our experiences from three projects have shown that using the robot in dementia research requires careful coordination, planning, and adjustments throughout the entire research process.

#### Organize with champions

Staying organized and planning ahead is crucial to avoid obstacles and provide clarity for all involved in research. Scheduling conflicts and lack of preparation led to issues in our research projects. Coordinating with designated “champions” (staff members who support the research) at the research sites is essential for smooth operations, as they have a better understanding of how the site operates. Additionally, planning should start early on in the research process to address any concerns staff or residents may have about using the technology.

#### Blend strategies

The research team should blend various research strategies to explore how to fully utilize the robot for research. For example, in our projects, using the robot in regular weekly research meetings helped patient and family partner become more comfortable and proficient with the robot by utilizing all its features. Additionally, regular meetings provide an opportunity to establish trust and foster teamwork, leading to a more effective partnership.

#### Offer timely technical assistance

Technical challenges may occur during research set-up or the data collection process when using the robots. It’s important to anticipate these issues and have support ready to offer timely technical assistance as soon as possible when necessary. This will ensure that data collection is not hindered. Additionally, lack of support can lead to researchers becoming discouraged by technical difficulties, potentially discouraging the use of the technology even though it may have advantages in that context.

#### Tailor training to meet individual needs

Training should be tailored to meet the needs of staff in LTC and researchers and should be completed before research begins. Each research site will have different operating schedules and workflow which must be taken into account. Furthermore, every researcher will have a different background and level of familiarity with this technology; some may require more support through training and practice than others, which should be accounted for when preparing for research.

### Implications

We have explored the use of telepresence robots as a remote research tool. Other studies have documented the creation of robotic platforms for use in research [37], but have not thoroughly examined telepresence robots as a research tool for participatory studies and their potential compared to other platforms/methods.

With the development of new virtual tools, there has been a growing number of research studies using web conferencing and remote platforms [38, 39]. Telepresence robots present a promising alternative to in-person interviews, complementing commonly used options such as Zoom and phone interviews. One notable advantage of telepresence robots over other virtual conferencing tools is the increased physical presence they provide, which can enhance engagement and overcome a common limitation (engagement in participation) of other virtual platforms [40, 41]. Our use of telepresence robots also showed improved engagement during interviews, which is often lacking in virtual research [40]. Participants found the technology easy to use and intuitive. The need for virtual research options has become increasingly pressing due to infection risks, time, cost, shortage of staff in healthcare [3]. As the world becomes more interconnected, virtual research options are likely to become even more important in the future. Virtual options are often used in situations where in-person administration is feasible, for benefits such as saving travel time and enabling easier connections for people in distant locations. Therefore, telepresence robots may serve as one of many solutions to further enable virtual research by providing benefits over other virtual platforms.

Using telepresence robots for research also has drawbacks, such as the need for technical support and the prevalence of technical difficulties, similar to other virtual methods [39, 41]. Due to their novelty, more training must be provided to researchers and staff to operate and maintain the robot effectively. Prevalent understaffing problems in healthcare may also limit the effectiveness of telepresence robots, as it can be difficult to find time to provide training and engage staff in the research [1]. Staff are also needed to transport the robot to the designated space for interviews and setting up of the robot in the room. Additionally, the cost of telepresence robots may be a potential limiting factor for some organizations; the telepresence robots used in our research study cost about 4000 USD each. The recent shift to virtual platforms for research has had a negative impact on patient and family partner engagement [42]. However, their involvement in research is crucial to ensure relevance and acceptance of research; providing additional, more user-friendly options is important to enable meaningful engagement of patient and family partners in research. Across our three studies, the use of telepresence robots allowed us to involve our patient and family partners in research through virtual interviews and design workshops, which would not have been possible otherwise due to in-person visitations. Telepresence robots increased patient engagement across our research projects; patient partners often described telepresence robots as the closest thing to being in-person and preferred using them once they became comfortable with the technology. PPI is more difficult now due to reported lower engagement through virtual platforms [42], however, telepresence robots seem to allow for increased engagement of patient partners in research. Even though visitation restrictions may not always exist, telepresence robots allow for increased engagement of patient partners virtually, regardless of where they may be located in the world. This increased accessibility has the potential to improve research outcomes in future work.

Our study primarily focuses on conducting participatory research using the robot in LTC homes. With careful consideration of barriers and opportunities, the robot has the potential to be used in many areas of research. In addition to one-on-one interviews and virtual workshops, the robot could be used for ethnographic research and focus groups. Further research should be conducted to explore its potential benefits in these types of studies. Observational research is currently difficult to perform in healthcare settings due to visit limitations, especially as these studies would require prolonged engagement. Telepresence robots, however, allow for more frequent and longer visits without compromising public health. Additionally, focus groups currently conducted through other virtual platforms such as Zoom would benefit from the increased physical presence and environmental engagement provided by telepresence robots [40, 41]. Furthermore, any research that limits the number of researchers that can be present would benefit greatly from using this technology. While these applications have yet to be investigated, our experience with telepresence robots suggests they will provide benefits over current methods of virtual research such as Zoom.

## Strengths and limitations

One strength of this self-study is the involvement of a patient partner in the whole process, from planning, design, data collection, analysis and dissemination of research findings. Our patient partner contributed to giving direct guidance throughout the entire research process. The regular research meetings embedded with team reflection helped to build trust and relationships, which provided a safe environment for teamwork. Some limitations of this study need to be acknowledged. The researchers who used the telepresence robots, along with this paper’s authors, are all members of a research lab based at the University of British Columbia that focuses on technology and innovation in senior care. These individuals may have been biased toward the robot because of their involvement of robots in research projects. Furthermore, the sample size of individuals who we interviewed about using the robot was small and concentrated in one geographical location, an urban Canadian city. Future research should include a wider range of regions, including rural areas.

## Conclusions

This study offers practical insights into using telepresence robots as a safe and innovative tool for conducting research remotely to include patient partners and people with dementia in research. Future research should apply more creativity and flexibility in adopting technology to expand possibilities for involving people with dementia in research. Our acronym ROBOT offers five actionable recommendations that inspire future research to use telepresence robots as a tool for research: Realign to adapt, Organize with champions, Blend strategies, Offer timely technical assistance, and Tailor training to individual needs.

## Data Availability

Dr. Lillian Hung may be contacted if someone would like to request the data.

## Abbreviations

LTC: Long-term care
POR: Patient-oriented research
SPOR: Strategy for patient-oriented research
CIHR: Canadian Institutes of Health Research
PAR: Participatory action research
